# Comparative analysis of core and perfusion lesion volumes between commercially available computed tomography perfusion software

**DOI:** 10.1177/23969873221135915

**Published:** 2022-11-18

**Authors:** Olli P Suomalainen, Nicolas Martinez-Majander, Gerli Sibolt, Katariina Bäcklund, Juha Järveläinen, Antti Korvenoja, Marjaana Tiainen, Nina Forss, Sami Curtze

**Affiliations:** 1 Department of Neurology, Helsinki University Hospital and Clinical Neurosciences, University of Helsinki, Finland; 2 Department of Neuroradiology, Helsinki University Hospital and Clinical Neurosciences, University of Helsinki, Finland; 3 Department of Neuroscience and Biomedical Engineering, Aalto University, Finland

**Keywords:** Ischemic stroke, ischemic core, penumbra, CT perfusion

## Abstract

**Introduction:**

Computed tomography perfusion (CTP) imaging has become an important tool in evaluating acute recanalization treatment candidates. Large clinical trials have successfully used RAPID automated imaging analysis software for quantifying ischemic core and penumbra, yet other commercially available software vendors are also on the market. We evaluated the possible difference in ischemic core and perfusion lesion volumes and the agreement rate of target mismatch between OLEA, MIStar, and Syngo.Via versus RAPID software in acute recanalization treatment candidates.

**Patients and methods:**

All consecutive stroke-code patients with baseline CTP RAPID imaging at Helsinki University Hospital during 8/2018–9/2021 were included. Ischemic core was defined as cerebral blood flow <30% than the contralateral hemisphere and within the area of delay time (DT) >3s with MIStar. Perfusion lesion volume was defined as DT > 3 s (MIStar) and T_max_ > 6 s with all other software. A perfusion mismatch ratio of ⩾1.8, a perfusion lesion volume of ⩾15 mL, and ischemic core <70 mL was defined as target mismatch. The mean pairwise differences of the core and perfusion lesion volumes between software were calculated using the Bland-Altman method and the agreement of target mismatch between software using the Pearson correlation.

**Results:**

A total of 1606 patients had RAPID perfusion maps, 1222 of which had MIStar, 596 patients had OLEA, and 349 patients had Syngo.Via perfusion maps available. Each software was compared with simultaneously analyzed RAPID software. MIStar showed the smallest core difference compared with RAPID (−2 mL, confidence interval (CI) from −26 to 22), followed by OLEA (2 mL, CI from −33 to 38). Perfusion lesion volume differed least with MIStar (4 mL, CI from −62 to 71) in comparison with RAPID, followed by Syngo.Via (6 mL, CI from −94 to 106). MIStar had the best agreement rate with target mismatch of RAPID followed by OLEA and Syngo.Via.

**Discussion and conclusion:**

Comparison of RAPID with three other automated imaging analysis software showed variance in ischemic core and perfusion lesion volumes and in target mismatch.

## Introduction

Computed tomography perfusion (CTP) imaging has become an important selection tool for evaluating acute stroke patients in recent years.^1–5^ Although comprehensive diffusion-weighted magnetic resonance imaging (MRI-DWI) offers the most accurate estimation of early infarction and ischemic core, the main downsides of MRI are low availability outside comprehensive stroke centers, safety issues with implants and possible metallic objects in patients, access to MRI in emergency settings, a possible longer scanning time, and movement artifacts with agitated patients. This makes CTP more feasible in many centers.^2,3,6^

CTP and especially RAPID automated imaging analysis are increasingly used as selection tools for the endovascular treatment (EVT) of large vessel occlusion (LVO) in a 6-to-24-h time window, for intravenous thrombolysis (IVT) in a 4.5–9 h time window after stroke onset and with wake-up stroke patients.^7–14^ CTP is also often used when there is suspicion of a stroke mimic or to help in clinical decision making if there are relative contraindications for IVT or EVT.^2^

CTP imaging analysis produces brain perfusion maps that indicate several parameters, including cerebral blood volume (CBV), cerebral blood flow (CBF), mean transit time (MTT), and time to peak of the residual function of an injected contrast agent (*T*_max_).^2^ A T_max_ delay exceeding 6 s estimates critically hypoperfused brain tissue with RAPID automated imaging software, including both ischemic core and penumbra.^12,13^ RAPID software has been widely used in several large trials to estimate the volumes of ischemic core and perfusion lesion and several guidelines rely on these trials.^2,7–9^ Several studies have shown that ischemic core obtained from CTP automated imaging analysis software reliably predicts the final infarct volume estimated from comprehensive MRI-DWI.^2,10,12–16^ At present, several commercially available CTP automated imaging analysis software (e.g. MIStar, OLEA, and Syngo.Via) exist on the market, some of which have been compared with RAPID with high agreement.^13,17–26^ Performance of the commonly used CTP software packages (MIStar, OLEA, and Syngo.Via) remain unclear when compared with RAPID in large clinical trials among stroke code candidates.

We aimed to evaluate the possible difference in automatically calculated volumes of ischemic core and perfusion lesion and the agreement of target mismatch between RAPID and three other commercially available CTP automated imaging analysis software (OLEA, MIStar, and Syngo.Via) in acute recanalization treatment candidates imaged with CTP in a single comprehensive stroke center.

## Patients and methods

A single-center, retrospective analysis of imaging findings of all consecutive acute stroke patients (Stroke Code) was performed from August 2018 to September 2021 at Helsinki University Hospital (HUS) based on the Helsinki stroke quality registry (HSQR).

We use non-enhanced computed tomography (NCCT) and computed tomography angiography (CTA) as the first-line imaging modalities for stroke code patients at HUS. CTP is required per protocol in the extended time window for IVT (after 4, 5–9 h of symptom onset and with wake-up stoke patients) and for EVT (after 6–24 h of symptom onset) for visualizing the ischemic core and perfusion lesion, as guided by current American Heart Association guidelines and our local guidelines.^9^ In the early time window CTP is performed on decision of the treating stroke neurologist.

Inclusion criteria were as follows: suspicion of an acute ischemic stroke within the extended time-window for potential acute recanalization therapy (IVT, EVT, or both) and a successful CTP RAPID imaging performed at baseline. Demographical (sex, age) and clinical parameters (site of acute vessel occlusion), along with a National Institute of Health Stroke Scale (NIHSS) score were registered at baseline. Patients evaluated as acute stroke code candidates with suspicion of an acute ischemic stroke at baseline were considered stroke mimics after comprehensive clinical evaluation and (baseline and follow-up) imaging if no signs of acute ischemia were found or a more likely cause other than acute ischemia for admission was diagnosed.

### Imaging protocol

CTP was performed on a Siemens Edge or Force (Siemens, Erlangen, Germany) 128-section scanners. The following parameters were used for CTP acquisition: a slice thickness of 5 mm, a collimator of 32 mm ×1.2 mm, 70 kVp, 135 mA with a total coverage of 100 mm and image coverage of 114 mm and with a radiation dose of 76.4 mGy (milligray) and 1129 mGy * cm. The imaging plane was parallel to the floor of the anterior cranial fossa starting just above the orbits. Thirty cycles were obtained with a total scan time of 46 s. The CTP images were sent without delay immediately after acquisition to RAPID, OLEA, MIStar, and Syngo.Via automated imaging analysis software without any user interaction or forcing classification of affected side to quantify ischemic core and perfusion lesion volumes.

### Automated imaging analysis software on computed tomography perfusion

RAPID and MIStar automated imaging analysis software were used to include patients from August 2018 to the end of the study. The OLEA software was available from August 2018 to November 2019 and Syngo.Via software from November 2020 to the end of the study period. The differences between automated imaging analysis software when assessing ischemic core and perfusion lesion volume are shown in Supplemental Table s1. The perfusion lesion is the critically hypoperfused area (prone to infarct), including ideally both penumbra and ischemic core. However, as perfusion lesion and ischemic core are acquired from different parameters, it is technically possible, but biologically not plausible, to have an ischemic core remote form the perfusion lesion. However, MIStar ignores an ischemic core outside the perfusion lesion volume.

**Figure 1.**
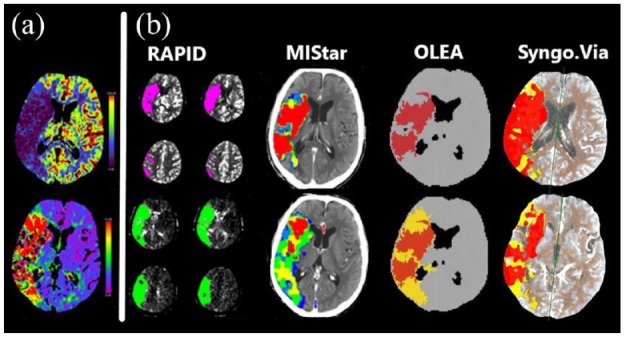
Computed tomography perfusion imaging in a 72-year-old male with occlusion of the right internal carotid artery and proximal M1-branch of the middle cerebral artery (tandem occlusion): (a) cerebral blood flow (upper row) and time-to-maximum (lower row) and (b) ischemic core volumes (upper row, red or magenta) and perfusion lesion volumes (lower row, green and yellow) by different automated imaging analysis software packages.

Figure 1 illustrates ischemic core and perfusion lesion volumes estimated with each automated imaging software in a 72-year-old male with occlusion of both the internal carotid artery and the proximal middle cerebral artery (tandem occlusion).

RAPID (iSchemaView Inc, Menlo Park, CA) uses a deconvolution method, that is thought to be delay insensitive^26^ and defines ischemic core as regions with a relative CBF < 30% than the contralateral hemisphere and perfusion lesion volume as a delayed arrival of an injected contrast agent bolus with a maximum of the residue function exceeding 6 s (T_max_ >6 s).^27^

AutoMIStar (Apollo Medical Imaging Technology) automatically generates delay time (DT), absolute CBF, CBV, and MTT maps using delay and dispersion-corrected singular value deconvolution (dd-SVD).^24^ The study by Lin et al.^19^ confirmed that a delay time of >3 s was the optimal threshold for perfusion lesion and a relative CBF of <30% within the area of delay time >3 s was the optimal threshold for ischemic core. Delay time >3 s has been shown to perform equally well as T_max_ >6 s.^18^

Olea Sphere (OLEA medical Inc., La Ciotat, France) uses an SVD postprocessing method like RAPID and uses CBF < 30% than the contralateral hemisphere and T_max_ >2 s which is used to rule out an old infarct and these matches with ischemic core by RAPID software as in our study. The perfusion lesion volume is defined as T_max_ >6 s.^20^

Syngo.Via (CT Neuro Perfusion VB40, Siemens Healthcare, Erlangen, Germany) relies on a deconvolution model with a delay-insensitive algorithm as well as on interhemispheric comparison.^15^ The side with the highest time to drain is automatically characterized as the lesion side and the contralateral side is used as a reference for relative values. Ischemic core is defined by a relative CBF < 30% than the contralateral hemisphere and perfusion lesion volume by T_max_> 6 s.

Criteria for target mismatch were met if (a) the volume of perfusion lesion divided by ischemic core volume (mismatch ratio) was 1.8 or more, (b) the perfusion lesion volume was ⩾15 mL, and (c) the ischemic core volume was <70 mL.^9^ Outliers with a difference in ischemic core or perfusion lesion volumes of >300 mL between the two software visually inspected for quality and plausibility. Perfusion maps suggesting diffuse ischemia in both hemispheres or not bound to vascular territories, or outside the brain area were classified as artifactual.

### Statistics

Descriptive statistics were performed using SPSS, version 25.0 (IBM Corp., Armonk, NY, USA). The Shapiro-Wilk test was used to assure normality of the continuous variables. Categorical variables are presented as absolute values and percentages, continuous variables as the mean ± standard deviation (SD) if normally distributed or as the median (interquartile range, IQR) if skewed. Pearson’s correlation coefficient was calculated to estimate the correlation of ischemic core, perfusion lesion, and agreement on target mismatch between RAPID and other CTP imaging analysis software. The mean pairwise differences between RAPID and other software with corresponding 95% limits of agreement (confidence interval, CI) for both core volume and perfusion lesion volume estimates were calculated using the one-sample T-test.

Bland-Altman plots were used to illustrate the distribution of the difference in volumetric measurements (mL) between RAPID and other CTP software. The Bland-Altman plots enable visual assessment of the mean difference in values obtained between the paired measurements, data scatter, and the relationship between the magnitude of difference and size of measurement.^28^ The horizontal lines above and below the difference line represent 95% limits of agreement of the core volume and perfusion lesion volume and are defined with limits of agreement = difference ± 1.96 SD.

## Results

### Study population

**Figure 2.**
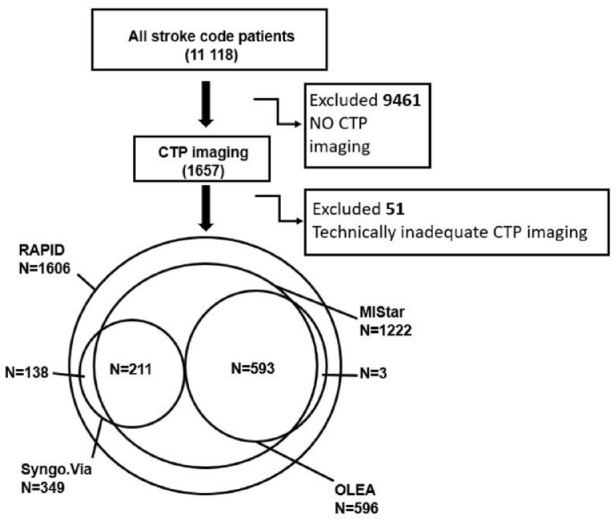
Flowchart of the patients in study including a Venn diagram of RAPID cohort and MIStar, OLEA and Syngo.Via cohorts. CTP: computed tomography perfusion imaging.

We screened 11 118 consecutive acute stroke code patients (Figure 2). Of those, 1657 (15%) patients had baseline CTP imaging with RAPID software. Fifty-one (3%) patients were excluded due to inadequacy in image acquisition (poor or absent bolus or excessive patient motion) that led to missing RAPID perfusion maps. Of the 51 excluded patients with missing RAPID perfusion maps, only one patient had artifact-free MIStar and OLEA perfusion maps. Among the 1606 patients with successful RAPID scans, scans were not successful in 42 patients with MIStar software, in 19 patients with OLEA and in 60 patients with Syngo.Via software. The general server of MIStar software was out of order or under service several (days) during study inclusion period resulting in missing perfusion scans during these periods.

The final cohort consisted of 1606 patients with CTP RAPID perfusion maps, 1222 (76%) of which had with MIStar, 596 (37%) had OLEA, and 349 (22%) had Syngo.Via perfusion maps available These three cohorts were used for head-to-head comparison (RAPID vs MIStar, RAPID vs OLEA, and RAPID vs Syngo.Via; Figure 2). Noteworthy, not all four software programs were in use at the same time. The overlapping cohort of three software was used for analysis of 593 (37%) patients (RAPID, MIStar, and OLEA) and of 211 (13%) patients for the three other software (RAPID, MIStar, and Syngo.Via). Baseline characteristics (Table 1) were similar for all cohorts although the Syngo.Via cohort had fewer patients with IVT and EVT and more patients with wake-up stroke than the OLEA and MIStar cohorts.

**Table 1.** Characteristics of all patients with RAPID imaging and the cohorts with additional MIStar, OLEA, and Syngo.Via automated imaging analysis software.

	RAPID	MIStar	OLEA	Syngo.Via
	*N* = 1606	*N* = 1222	*N* = 596	*N* = 349
Age in years, mean (SD)	66 (±15)	66 (±15)	66 (±15)	66 (±14)
IVT alone	309 (20)	251 (21)	113 (19)	54 (15)
IVT + EVT	131 (8)	92 (8)	50 (8)	26 (7)
EVT alone	146 (9)	133 (11)	72 (12)	28 (8)
Occlusion site (EVT), n				
MCA (M1)	116	84	47	17
ICA + *M*1 (tandem)	21	19	9	5
Basilar/vertebralis	21	19	8	2
other	50	36	15	13
Unknown time of symptom onset but last seen well,*	813 (51)	614 (50)	293 (49)	184 (53)
Known time of symptom onset	793 (49)	608 (50)	303 (51)	165 (47)
Time from symptom onset to imaging in patients with known time of symptom onset, minutes	122 (75–22)	124 (75–238)	127 (75–248)	110 (77–205)
Wake-up stoke	435 (27)	327 (27)	150 (25)	108 (31)
Stroke or TIA	1349 (84)	1024 (84)	487 (82)	303 (87)
Stroke Mimic	257 (16)	198 (16)	109 (18)	46 (13)
Stroke mimic IVT	2 (0)	1 (0)	1 (0)	0 (0)
CTP after initiation of IVT	89 (20)	66 (19)	40 (25)	13 (16)
Baseline NIHSS	5 (3–10)	5 (3–10)	5 (2–10)	5 (2–10)

IVT: intravenous thrombolysis; EVT: endovascular treatment; MCA: middle cerebral artery; ICA: internal carotid artery; TIA: transient ischemic attack; Stroke mimic: no signs of acute ischemia after comprehensive clinical evaluation and imaging nor transient ischemic attack; NIHSS; NIH Stroke Scale.

Data are n (%) or median (interquartile range, IQR) unless otherwise stated.

* Unknown time from symptom onset but last seen well within the time window for acute recanalization treatment.

### Head-to-head comparison of RAPID with other automated imaging analysis software

**Figure 3.**
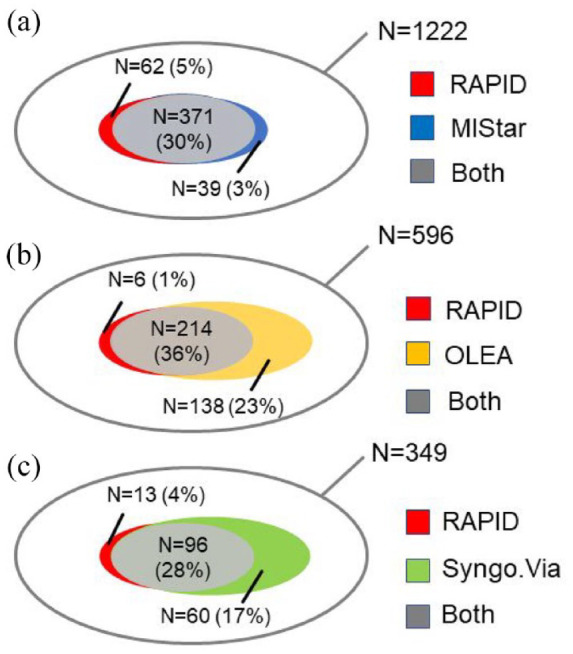
Venn diagram showing the proportion of patients who fulfill the target mismatch definition by each automated imaging analysis software. Target mismatch is defined as (a) volume of perfusion lesion divided by ischemic core volume (mismatch ratio) ⩾1.8, (b) a perfusion lesion volume of ⩾15 mL, and (c) a ischemic core volume less <70 mL by each software. The outer (gray) shell represents the compared cohort. Overlapping gray areas represent patients in whom both software classified a target mismatch. The non-overlapping color indicates the proportion of target mismatch found by one software but not the other.

Figure 3 depicts the number of patients with target mismatch identified by RAPID and other automated imaging analysis software. The proportion of cases was small, where a target mismatch was classified by RAPID only, but classified as no target mismatch by the software of comparison (MIStar 5%, OLEA 1%, and Syngo.Via 4%; Figure 3). However, for cases where RAPID classified absence of target mismatch while the software of comparison classified presence of target mismatch was small only for MIStar (3%), while larger for Syngo.via (17%) and OLEA (23%). Out of these patients with absent target mismatch with RAPID software, but present mismatch on the other software, 22% (OLEA) and 15% (Syngo.Via) were later classified as stroke mimics. Table 2 compares the results of each software with RAPID results. The number of patients with any perfusion lesion in stroke mimics versus RAPID software in each cohort are also shown in Table 2. OLEA and Syngo.Via classified perfusion lesion in stroke mimics more often and MIStar less often than RAPID software.

**Table 2.** Shows the difference in imaging results between MIStar, OLEA, and Syngo.Via compared with the RAPID cohort.

	RAPID	MIStar	OLEA	Syngo.Via
Target mismatch	546 (34)	410 (34)	352 (59)	156 (45)
Target mismatch classified with RAPID	546 (100)	371 (90)	214 (61)	96 (62)
Agreement (P§) on target mismatch with RAPID	1	0.82	0.60	0.59
Ischemic core volume mL	0 (0–2)	0 (0–7)	1 (0–4)	5 (0–19)
P§ ischemic core	1	0.92	0.80	0.43
Core difference mL (CI†)	NA	−2 (from −26 to 22)	2 (from −33 to 38)	−12 (from −116 to 92)
Perfusion lesion volume mL	0 (0–49)	0 (0–2)	25 (6–75)	15 (2–50)
Perfusion lesion >2 mL	771 (48)	598 (49)	530 (89)	265 (76)
P§ Perfusion lesion volume	1	0.88	0.73	0.76
Perfusion lesion volume difference mL (CI†)	NA	4 (from −62 to 71)	−18 (from −132 to 97)	6 (from −94 to 106)
Perfusion lesion versus RAPID	788 (49)	605 (50)	582 (98)	298 (85)
	NA	625 (51)	332 (56)	198 (57)
Ischemic core >70 mL versus RAPID	55 (3)	43 (4)	13 (2)	21 (4)
	NA	43 (4)	21 (4)	11 (3)
Perfusion lesion (>0 mL) in stroke mimics versus RAPID	61 (24)	31 (16)	52 (48)	7 (15)
	NA	52 (26)	40 (37)	37 (80)

Data are n (%) or median (interquartile range, IQR) unless otherwise stated.

C†: 95% limits of agreement by Bland-Altman, confidence interval; IQR: interquartile range; P§: Pearson’s Rank; NA: not applicable.

The agreement on target mismatch between MIStar and RAPID software was very strong (Pearson correlation = 0.92, P). MIStar classified the presence of large ischemic cores (>70 mL) similarly with RAPID (both 4%) while the presence of perfusion lesion volume was larger (51% vs 50%). The agreement on target mismatch between OLEA and Syngo.Via software versus RAPID software was strong (*p* = 0.60 and 0.59, respectively). Out of the 596 patients with OLEA and RAPID perfusion maps, OLEA showed fewer large ischemic cores (2% vs 4%, respectively) than RAPID but classified the presence of perfusion lesion volume more often than RAPID (98% vs 56%, respectively). Similarly, out of the 349 patients in Syngo.Via cohort, Syngo.Via classified both large ischemic cores (4% vs 3%, respectively) and presence of perfusion lesion volumes (85% vs 57%, respectively) more often than RAPID software.

**Figure 4.**
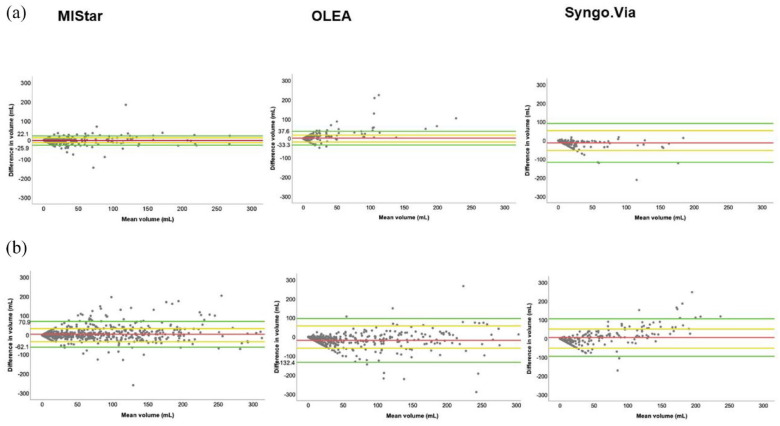
Bland-Altman plots of the differences in ischemic core volume (a) and in perfusion lesion volume (b) between MIStar, OLEA, and Syngo.Via software and RAPID software showing mean volume (milliters, mL) of two software in the *x*-axis and difference in volume in the *y*-axis (mL). The green horizontal lines above and below the difference line (red) represent 95% limits of agreement (confidence interval) of core volume and perfusion lesion volume and are defined with limits of agreement = difference ± 1.96 SD which is illustrated as yellow lines (±1 SD). For illustrative reasons, the difference in volume between RAPID and other software is limited to ±300 mL for core and perfusion lesion volume. The number of patients with more than ±300 mL difference not shown in Figure 4 but provided in Supplemental Material with whole scale are as follows; ischemic core volume (None with MIStar and OLEA and two with Syngo.Via software) and perfusion lesion volume (Two patients with MIStar and Syngo.Via and five with OLEA software).

Figure 4 visualizes with Bland-Altman plots the volume differences between ischemic core and perfusion lesion volume (±1 SD) to evaluate agreement of volumes of ischemic core and perfusion lesion with MIStar, OLEA, and Syngo.Via in comparison with RAPID. Differences in ischemic core and perfusion lesion volumes are restricted to ±300 for illustrative reasons. Supplemental Figure s2 shows the difference and mean for ischemic core and perfusion lesion volumes on the whole scale and perfusion parameters of individual patients with a more than ±300 mL difference in core and/or perfusion lesion volumes between RAPID and other CTP software.

MIStar gave smaller estimates for ischemic core volume (±1 SD 12 mL) and a larger estimate for perfusion lesion volume (±1 SD 34 mL). OLEA gave a larger estimate for ischemic core volume (±1 SD 18 mL) and a smaller estimate for perfusion lesion volume (±1 SD 58 mL) compared with RAPID software. Syngo.Via, like MIStar, gave smaller estimate for ischemic core volume (±1 SD 53 mL) and a larger estimate for perfusion lesion volume (±1 SD 51 mL) compared with RAPID software.

As RAPID and MIStar software usually do not report very small perfusion lesions (⩽2 mL) in contrast to OLEA and Syngo.Via software, we made an additional comparison of the volume of perfusion lesions >2 mL in each cohort. A perfusion lesion exceeding 2 mL was found with OLEA software in 89% of patients followed by Syngo.Via (76%), MIStar (49%), and finally RAPID (48%).

### Patient comparison with three perfusion software

A total of 593 patients had three simultaneous perfusion maps from RAPID, MIStar, and OLEA software available (Supplemental Figure s3). We found agreement on existence of a target mismatch in 37% of the cohort and agreement on absence of a target mismatch in 31% for all three software, while there was disagreement in 32% of the cases.

A total of 211 patients had three perfusion maps from RAPID, MIStar and Syngo.Via software available (Supplemental Figure s3). We found agreement on existence of a target mismatch in 27% of the cohort and agreement on absence of a target mismatch in 49% for all three software, while there was disagreement in 24% of the cases.

## Discussion

RAPID software has successfully been used in several large clinical trials, and it has predicted final infarct volume at follow-up imaging after EVT.^7–9,14^ Our aim was to compare commercially available CTP software head-to-head with RAPID software. Our results showed only small differences in ischemic core volumes between the software, whereas volume differences of the perfusion lesion were larger. MIStar classified target mismatch with a similar rate when compared with RAPID software, whereas both OLEA and Syngo.Via classified target mismatch more frequently than RAPID. The results of the head-to-head comparison did not differ between individual software in the subcohort of patients with perfusion maps from three vendors.

In general, our results are in line with previous studies comparing RAPID with other commercially available CTP software.^14,17–26^ The differences in ischemic core volume and perfusion lesion volume between RAPID and MIStar software were small in our study. Biswas et al.^23^ and Park et al.^26^ have previously also shown that RAPID and MIStar exhibit a small difference in ischemic core volume estimates and a larger difference in perfusion lesion volume estimates especially at small volumes and the agreement of RAPID and MIStar on these estimates was higher at larger volumes.

The study by Psychogios et al., comparing RAPID software with both OLEA and Syngo.Via, demonstrated differences in ischemic core estimates and especially in perfusion lesion volume estimates between these software. Psychogios et al.^21^ showed that the ischemic core volume estimate for OLEA was smaller but rather similar with RAPID, while there were relevant and a larger difference between RAPID and Syngo.Via software. In our study, the correlation of ischemic core between OLEA and RAPID software was strong but only moderate with Syngo.Via. We also found a similar core difference between RAPID and OLEA as in the study by Psychogios et al. (2 vs 3 mL). The smaller difference in perfusion lesion volume between RAPID and OLEA software (41.9 vs 25 mL) and in ischemic core volume between RAPID and Syngo.Via software (30 vs 12 mL) in our study compared with the study by Psychogios et al. may be due to larger sample size in our study.

Syngo.Via software has previously shown larger ischemic core volume estimates compared with RAPID using similar settings in a small cohort in a subset of MR CLEAN trial patients.^17^ Bathla et al. suggested a lower rCBF (<20%) than in our study, and T_max_ > 6 s with Syngo.Via software provides high correlation for both ischemic core and for perfusion lesion volume in comparison with RAPID software.^29^ Our results are in line with these studies, as Syngo.Via software showed largest difference of ischemic core in comparison with RAPID software. In our study, the correlation of ischemic core volume between RAPID and Syngo.Via software was only moderate, but the same threshold for CBF (<30%) was used with both Syngo.Via and RAPID software. Although we did not have follow-up infarct volumes, a recent study by Muehlen et al.,^22^ showed that the highest correlation between ischemic core volume and follow-up infarct between RAPID and Syngo.Via software in fully recanalized EVT patients was achieved indeed at different rCBF thresholds (<38% and <25% for the RAPID and Syngo.Via software, respectively).

Xiong et al. have previously shown that main drawback of RAPID is its numerically higher failure rate to detect ischemic brain regions compared with OLEA. Nevertheless, RAPID had a better accuracy for ischemic core volume and infarcts ⩾70 mL when compared with MRI-DWI infarct volume in this study.^20^ In our study, OLEA classified less and Syngo.Via more frequently large cores (>70 mL) than RAPID software. Both OLEA and Syngo.Via software classified also target mismatch, perfusion lesion and ischemic core lesion in stroke mimics more often than RAPID software. Whether the perfusion lesions detected with RAPID or other CTP software represent true ischemia, oligoemia, or artifacts cannot be answered in our study setting.

Austein et al.^14^ showed that RAPID had the highest precision and a good predictive accuracy for final infarct volume especially in early and fully recanalized EVT patients in comparison with Phillips and Syngo.Via software. In the study by Austein et al., RAPID also showed less overestimation of follow-up infarct volume in EVT patients and predicted perfusion lesion volume better in nonsuccessfully recanalized patients while Syngo.Via software significantly overestimated the perfusion lesion volume. Both resulted in false-positive estimates for a target mismatch profile and a respective large follow-up infarct volume (<70 mL). This could be one possible explanation for our results as Syngo.Via classified target mismatch more often than the RAPID software, and the estimates for perfusion lesion volume was larger than with the RAPID software. The number of patients in the study by Austein et al. was lower than in our Syngo.Via cohort and follow-up imaging took place between 24 h and 8 days which could induce variance in follow-up infarct estimation. Finally, the study by Gunasekera et al.^25^ has previously shown that delay- and dispersion corrected single-value decomposition of MIStar correlates better with MRI-DWI follow-up infarct volume than delay and dispersion insensitive deconvolution does with RAPID software in EVT patients with successful recanalization. This suggests that RAPID software may overestimate large ischemic cores when compared with MIStar which may lead to patient exclusion from EVT based on core volume selection. Despite this, our study showed high volumetric agreement in ischemic core volume between MIStar and RAPID software and the number of large cores (>70 mL) were similar with both software.

The major strength of our study is the large number of acute stroke code candidates imaged with CTP and the high proportion of patients treated with acute recanalization treatments (IVT, EVT, or both). Automated imaging analysis software were compared head-to-head in a clinical setting with subgroup analyses of patients with three perfusion software. The decision to proceed to acute recanalization treatment relied on current guidelines of acute recanalization treatment, which are based on large clinical trials.^7–9^ Patients who turned out to be stroke mimics were not excluded from the study, as they form an important group for CTP imaging in a clinical setting. All included patients were imaged with CTP solely at HUS, increasing the homogeneity of the study cohort.

The major limitation of our study is its retrospective nature. We did not have a consecutive cohort of patients with all four software vendors due to the OLEA software license expiring in September 2019 during the study inclusion period and failure of the Syngo.Via software to provide volumetric measures from raw CTP data from the RAPID, OLEA and MIStar cohorts before software upgrade in November 2020. RAPID, like other CTP software, may sometimes also underestimate or overestimate ischemic core volumes sand perfusion lesion volumes in individual patients.^13^ We did not measure the baseline ischemic core volume with comprehensive MRI, nor did we have final infarct volume measurements to assess the accuracy of ischemic core estimation for each automated imaging analysis software which is a major limitation. However, the CBF < 30% threshold in CTP imaging used by RAPID software in our study is extensively validated for core measurement^30^ although on average it may underestimate rather than overestimate MRI-DWI lesion but has greater specificity for predicting DWI positive voxels compared to the rCBF < 38% threshold.^31^ In the study by Nogueira et al.^7^ and Albers et al.^8^ RAPID also overestimated less often final infarct volume compared to Syngo.Via software and in large clinical trials concerning acute recanalization treatment it has predicted final infarct volume accurately when compared to MRI-DWI in EVT patients. Acquiring ischemic core volumes with MRI-DWI in addition to CTP at baseline could on the other hand delay the decision of proceeding to possible recanalization treatment. The MRI-DWI scan would have to be performed timely very close to the CTP imaging at baseline for accurate measurements between MRI-DWI and CTP imaging as the core often evolves after imaging and recanalization treatments interfere with the ischemic core progression.

CTP imaging has become an important part of acute stroke care especially after 4.5 h of symptom onset as the large clinical trials showed the EVT and IVT efficacy in late presenting patients.^7–10^ Although several automated imaging analysis software are on the market, only RAPID has been successfully used in these trials. Although we showed a small core difference and a larger difference in the perfusion lesion volume between three automated imaging software and RAPID, the more liberal classification of perfusion lesion as target mismatch by different software can lead to variance in the number of patients included in acute recanalization treatment. Further clinical trials confirming clinically relevant CTP measures are required.

## Conclusions

A comparison of three commonly used automated imaging software with RAPID showed a small core difference and a larger difference in perfusion lesion volume resulting in variance with the agreement rate of target mismatch when compared with RAPID software.

## Supplemental Material

sj-docx-1-eso-10.1177_23969873221135915 – Supplemental material for Comparative analysis of core and perfusion lesion volumes between commercially available computed tomography perfusion software Click here for additional data file. Supplemental material, sj-docx-1-eso-10.1177_23969873221135915 for Comparative analysis of core and perfusion lesion volumes between commercially available computed tomography perfusion software by Olli P Suomalainen, Nicolas Martinez-Majander, Gerli Sibolt, Katariina Bäcklund, Juha Järveläinen, Antti Korvenoja, Marjaana Tiainen, Nina Forss and Sami Curtze in European Stroke Journal
