# Supplementary material for: Comparative analysis of core and perfusion lesion volumes between commercially available computed tomography perfusion software
Source: Eur Stroke J. 2022 Nov 18;8(1):259–67. doi: 10.1177/23969873221135915 (PMC10069177; doi:10.1177/23969873221135915)
Supplement: sj-docx-1-eso-10.1177_23969873221135915 – Supplemental material for Comparative analysis of core and perfusion lesion volumes between commercially available computed tomography perfusion software [file sj-docx-1-eso-10.1177_23969873221135915.docx]

Supplemental material

# Table sI.

| Ischemic core volume and perfusion lesion volume analysis with different automated imaging software package | | | | |
| --- | --- | --- | --- | --- |
|  | RAPID | MIStar | OLEA | Syngo Via |
|  |  |  |  |  |
| Deconvolution method | delay insensitive  deconvolution | dd-SVD* | SVD+ | SVD  (delay insensitive) |
|  |  |  |  |  |
| Ischemic core | Relative CBF<30%† | relative CBF <30%† within the area of delay time > 3 | Relative CBF<30%† | Relative CBF<30%† |
|  |  |  |  |  |
| Perfusion lesion (critical hypoperfusion) | Tmax6s† | delay time > 3 s | Tmax6s† | Tmax6s† |
|  |  |  |  |  |
| †CBF; cerebral blood flow, *dd-SVD; dispersion-corrected singular value deconvolution, +SVD; singular value deconvolution | | | | |

# Figure sI

Linear correlation of ischemic core volume and perfusion lesion volume (mL) in MIStar, OLEA and Syngo.Via cohorts in comparison with RAPID.


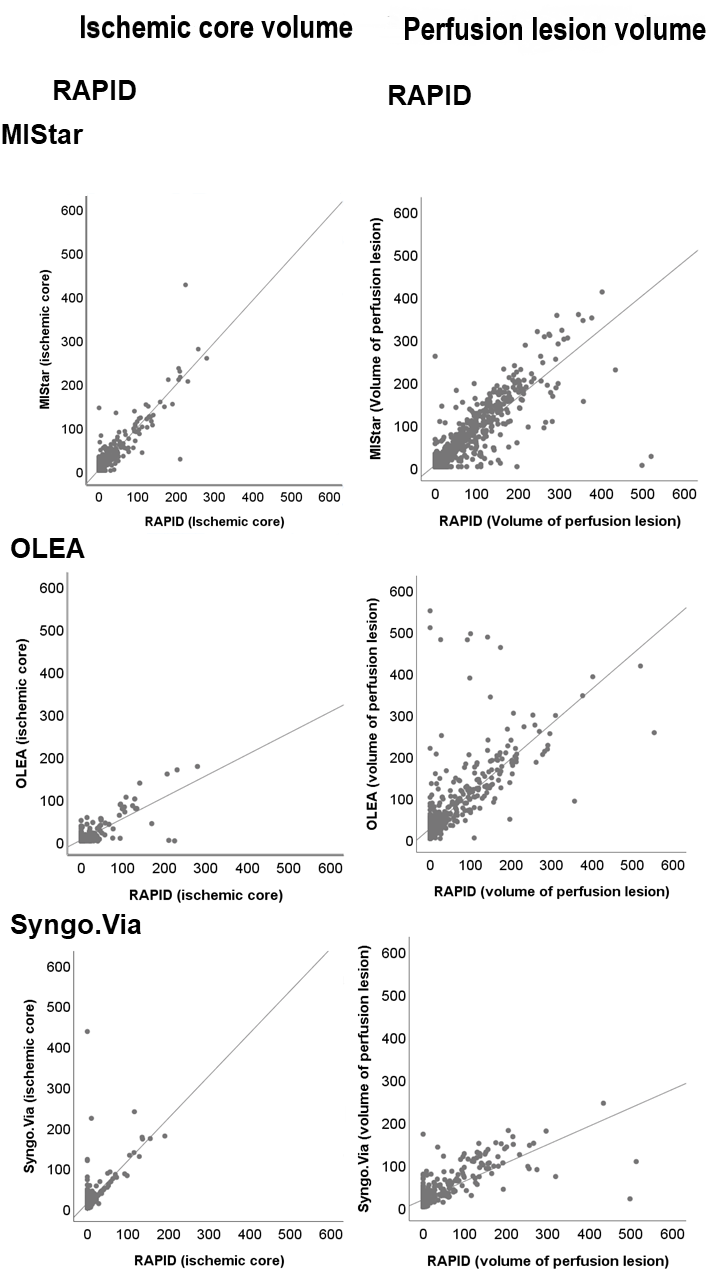


# Figure sII
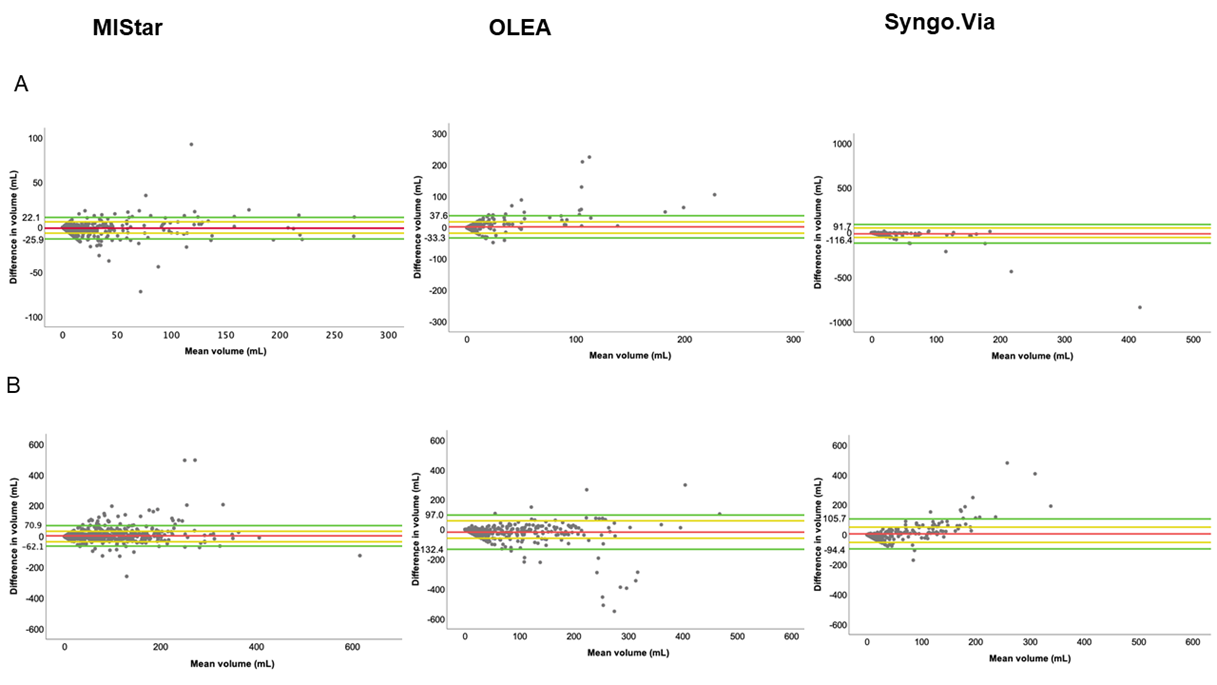


Bland–Altman plots of the difference of ischemic core volume (A) and in perfusion lesion volume (B) between MIStar, OLEA and Syngo.Via software and RAPID software in all patients with whole scale showing mean volume (milliters, mL) of two software in x-axis and difference in volume in y-axis (mL).

The green horizontal lines above and below the bias line (red) represent 95% limits of agreement of core volume and perfusion lesion volume and are defined with limits of agreement = bias ± 1.96 standard deviation which is illustrated as yellow lines (+/- 1 SD).

Outliers with a difference of more than 300 mL between the two software, were visually inspected for quality and plausibility. Perfusion maps suggesting diffuse ischemia in both hemispheres, or not bound to vascular territories or outside the brain area were classified as artifactual

# Difference of +/−300 m) in ischemic core volume between individual software and RAPID:

- MIStar and OLEA (none)
- Syngo.Via (two patients)
- Case 1 had ischemic core of 0 mL and perfusion lesion volume 319 mL by RAPID and 434 mL and 71 mL by Syngo.Via suggesting artifact by Syngo.Via.
- Case 2 had ischemic core of 0 mL and perfusion lesion volume lesion 0 mL by RAPID and 835 mL and 6mL by Syngo.Via suggesting artifact by Syngo.Via.

# Difference of +/−300 m) in perfusion lesion volume between individual software and RAPID:

*MIStar (two patients)*

- Case 1 had ischemic core of 6 mL and perfusion lesion volume 520mL by RAPID and 3mL and 24 mL by MIStar suggesting overestimation/failure by RAPID.
- Case 2 had ischemic core of 0 mL and perfusion lesion volume 498 mL by RAPID and 2 mL and 3 mL by MIStar suggesting overestimation/failure by RAPID.

*OLEA (five patients)*

- Case 1 had ischemic core of 211 mL and perfusion lesion volume 142 mL by RAPID and 1 mL and 485 mL by OLEA suggesting artifact by both software.
- Case 2 had ischemic core of 0 mL and perfusion lesion volume 92 mL by RAPID and 0 mL and 479 mL by OLEA suggesting artifact by OLEA.
- Case 3 had ischemic core of 15 mL and perfusion lesion volume 98 mL by RAPID and 0 mL and 494 mL by OLEA suggesting artifact by OLEA.
- Case 4 had ischemic core of 0 mL and perfusion lesion volume 26 mL by RAPID and 0 mL and 479 mL by OLEA suggesting artifact by OLEA.
- Case 5 had ischemic core of 0 mL and perfusion lesion volume 0 mL by RAPID and 0 mL and 508 mL by OLEA suggesting artifact by OLEA.

*Syngo.Via (two patients)*

- Case 1 had ischemic core of 0 mL and perfusion lesion volume 498 mL by RAPID and 57 mL and 18 mL by Syngo.Via suggesting artifact by both software.
- Case 2 had ischemic core of 116 mL and perfusion lesion volume 513 mL by RAPID and 237 mL and 106 mL by Syngo.Via suggesting artifact by both software

# Figure sII


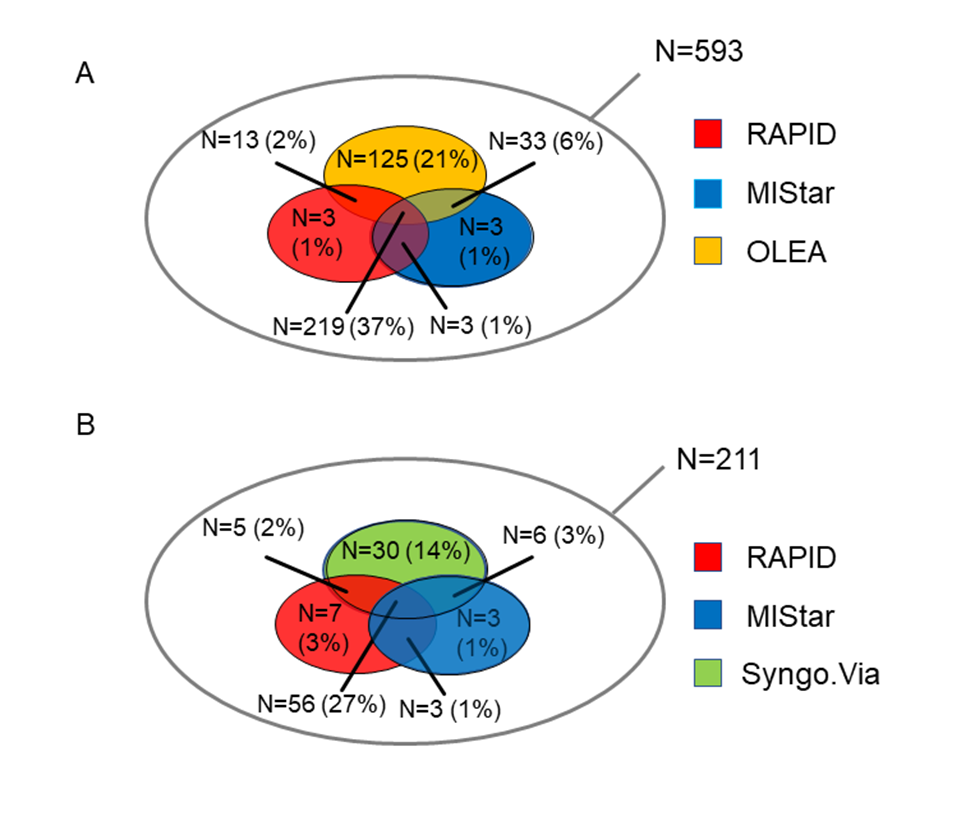


Venn diagram showing percentage of target mismatch of individual automated imaging analysis software in subcohorts with three different automated imaging analysis software A (RAPID, MIStar, and OLEA) and B (RAPID, MIStar and Syngo.Via).

Overlapping areas in the Venn diagram represent agreement on existence of a target mismatch of two or three software. Areas without overlap represent target mismatch of only one individual software and the white area represents cases where there was agreement of all three software on the absence of a target mismatch.
